# Enhancing the Solubility and Oral Bioavailability of Trimethoprim Through PEG-PLGA Nanoparticles: A Comprehensive Evaluation of In Vitro and In Vivo Performance

**DOI:** 10.3390/pharmaceutics17080957

**Published:** 2025-07-24

**Authors:** Yaxin Zhou, Guonian Dai, Jing Xu, Weibing Xu, Bing Li, Shulin Chen, Jiyu Zhang

**Affiliations:** 1 Key Laboratory of New Animal Drug Project of Gansu Province, Lanzhou 730050, China; zhouyaxin@caas.cn (Y.Z.); dai940910@163.com (G.D.); 82101221334@caas.cn (J.X.); pharm2005bl@126.com (B.L.); 2 Key Laboratory of Veterinary Pharmaceutical Development, Ministry of Agriculture, Lanzhou 730050, China; 3 Lanzhou Institute of Husbandry and Pharmaceutical Sciences, Chinese Academy of Agricultural Sciences, Lanzhou 730050, China; 4 College of Veterinary Medicine, Northwest A&F University, Xianyang 712100, China; 5 College of Science, Gansu Agricultural University, Lanzhou 730070, China; xuwb@gsau.edu.cn

**Keywords:** trimethoprim, PEG-PLGA, nanoparticles, bioavailability, pharmacokinetics

## Abstract

**Background/Objectives:** Trimethoprim (TMP), a sulfonamide antibacterial synergist, is widely used in antimicrobial therapy owing to its broad-spectrum activity and clinical efficacy in treating respiratory, urinary tract, and gastrointestinal infections. However, its application is limited due to poor aqueous solubility, a short elimination half-life (t_1/2_), and low bioavailability. In this study, we proposed TMP loaded by PEG-PLGA polymer nanoparticles (NPs) to increase its efficacy. **Methods:** We synthesized and thoroughly characterized PEG-PLGA NPs loaded with TMP using an oil-in-water (O/W) emulsion solvent evaporation method, denoted as PEG-PLGA/TMP NPs. Drug loading capacity (LC) and encapsulation efficiency (EE) were quantified by ultra-performance liquid chromatography (UPLC). Comprehensive investigations were conducted on the stability of PEG-PLGA/TMP NPs, in vitro drug release profiles, and in vivo pharmacokinetics. **Results:** The optimized PEG-PLGA/TMP NPs displayed a high LC of 34.0 ± 1.6%, a particle size of 245 ± 40 nm, a polydispersity index (PDI) of 0.103 ± 0.019, a zeta potential of −23.8 ± 1.2 mV, and an EE of 88.2 ± 4.3%. The NPs remained stable at 4 °C for 30 days and under acidic conditions. In vitro release showed sustained biphasic kinetics and enhanced cumulative release, 86% at pH 6.8, aligning with first-order models. Pharmacokinetics in rats revealed a 2.82-fold bioavailability increase, prolonged half-life 2.47 ± 0.19 h versus 0.72 ± 0.08 h for free TMP, and extended MRT 3.10 ± 0.11 h versus 1.27 ± 0.11 h. **Conclusions:** PEG-PLGA NPs enhanced the solubility and oral bioavailability of TMP via high drug loading, stability, and sustained-release kinetics, validated by robust in vitro-in vivo correlation, offering a promising alternative for clinical antimicrobial therapy.

## 1. Introduction

Trimethoprim (TMP), recognized as a sulfonamide antibacterial synergist, plays a pivotal role in antimicrobial therapy due to its broad-spectrum activity, minimal adverse effects, and significant post-antibacterial effect [1,2]. TMP exerts its antibacterial effect by specifically inhibiting dihydrofolate reductase, which prevents the reduction of dihydrofolic acid to tetrahydrofolic acid, thus blocking folate metabolism and augmenting the antibacterial activity of sulfonamides [3]. This mechanism underpins its widespread clinical application, particularly in the compound preparation co-trimoxazole (a combination of TMP and sulfamethoxazole), which is effective against respiratory infections caused by pathogens such as *Streptococcus pneumoniae* and *Haemophilus influenzae* [4,5]. Additionally, TMP demonstrates efficacy in treating urinary tract infections caused by *Escherichia coli*, as well as typhoid fever and gastrointestinal infections [6,7]. In China, TMP is classified as a Class A antimicrobial agent in the “National Basic Medical Insurance Drug List”, reflecting its substantial market potential and therapeutic value. In the veterinary field, TMP–sulfonamide combinations are extensively utilized to manage livestock and poultry infections, including swine colibacillosis and avian coccidiosis [8,9]. Despite its therapeutic potential, the clinical utility of TMP is hindered by poor aqueous solubility and compromised by irrational usage. From a pharmacokinetic perspective, TMP has a short elimination half-life (t_1/2_) and a mean residence time (MRT) in most mammals, resulting in low oral bioavailability [10]. Notably, the reduction of TMP release in stomach might be a potential way to achieve excellent oral bioavailability since TMP has maximum solubility in low pH (>1000 mg/mL in pH < 2) [11]. Consequently, it is essential to develop sustained release formulations that can enhance the stability and absorption performance of TMP.

Researchers have made efforts to explore various methods for enhancing the solubility of TMP. Garnero et al. and Li et al. prepared TMP complexes with β-cyclodextrin or hydroxypropyl-β-cyclodextrin, which significantly enhanced its aqueous solubility. However, due to the absence of direct animal experiments, the impact on its oral bioavailability remains uncertain [12,13]. Utilizing nano-delivery technology to improve drug solubility and enhance bioavailability serves as an effective strategy for optimizing therapeutic outcomes. Nano-antibacterial preparations, with the advantages of small size, high bactericidal efficacy, and low resistance induction, offer innovative approaches for the prevention and control of infectious diseases [14,15]. The poly (lactic-co-glycolic acid) (PLGA) loaded with TMP has been found to be highly suitable for the instillation therapy of urinary tract infections, while its potential as an oral drug remains unexplored [16]. Although a polyethylene glycol (PEG)ylated nanostructured lipid carrier represents a promising strategy for enhancing the oral delivery of TMP/sulfamethoxazole combinations, their limited drug LC (9.8%) poses significant challenges [10]. Studies have shown that insufficient drug loading in nanocarriers critically impairs their delivery efficiency by reducing nanoparticle adsorption on bacterial surfaces and weakening transmembrane permeability. This cascade effect leads to subtherapeutic intracellular drug concentrations below the minimum inhibitory concentration, ultimately diminishing antibacterial efficacy [17]. Therefore, the development of a new dosage with good drug LC, enhanced stability, and improved oral bioavailability is urgent for TMP.

Biodegradable polymeric NPs play a crucial role in enhancing the therapeutic properties of various drugs and bioactive compounds. Take PLGA, a functional polymeric organic compound approved by the FDA for drug delivery, for example, which decomposes into small molecules like carbon dioxide and water within the body and these are subsequently excreted via normal metabolic pathways [18]. However, studies have indicated that the hydrophobic surface of PLGA NPs makes them readily recognizable and bindable by plasma opsonin. This leads to their swift clearance by the mononuclear phagocytic system (MPS), significantly shortening their in vivo circulation time [19]. To address this issue, coating PLGA NPs with hydrophilic PEG has emerged as a strategic solution. PEG inhibits opsonin adsorption through steric repulsion effect and forms a dense hydrated brush layer on the NPs surface, rendering them invisible to the MPS and prolonging plasma circulation time [20]. As the first and most extensively studied surface modifier for long-circulating PLGA-based particles, PEG confers near-neutral zeta potentials, enhances steric stabilization in biological media, and improves the entrapment of insoluble drugs [21,22]. PEG-PLGA NPs achieve over 32% LC for the poorly soluble anti-fibrotic agent pirfenidone, while significantly enhancing drug stability and sustained-release kinetics [23]. Yao et al. demonstrated that PEG-PLGA not only improves the solubility and cellular uptake of isoliensinine but also enhances its distribution in critical tissues such as the heart, liver, lungs, and kidneys, ultimately boosting bioavailability [24]. Therefore, in light of the persistent challenges in improving the solubility and bioavailability of TMP, modifying TMP NPs with PEG-PLGA emerges as a novel and promising strategy. This approach holds the potential to overcome problems of low solubility, insufficient drug LC, and poor bioavailability of TMP, thereby offering a new avenue for optimizing its therapeutic effects.

**Figure 1:**
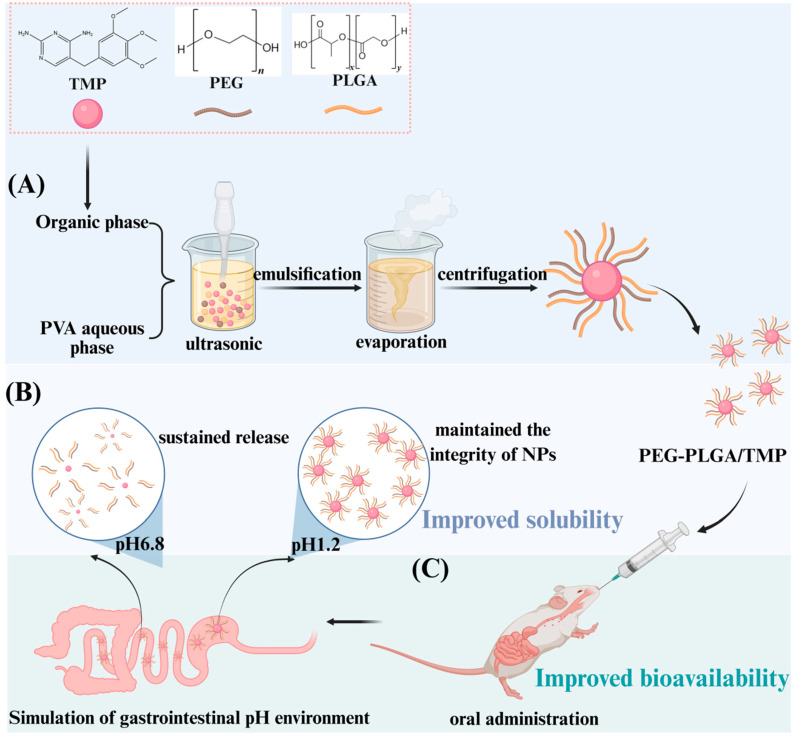
Schematic illustration of the formation process and applications of PEG-PLGA/TMP NPs. (**A**) Preparation of PEG-PLGA/TMP NPs via an emulsification evaporation method. (**B**) Release characteristics of PEG-PLGA/TMP NPs in simulated gastrointestinal pH environments. (**C**) Oral administration of PEG-PLGA/TMP NPs. We drew this picture using “BioRender”; we obtained their publishing permission, and the agreement number is “MT28BK9T1W”.

In this work, we encapsulated TMP within biodegradable PEG-PLGA blend NPs through an O/W emulsion solvent evaporation method to create PEG-PLGA/TMP NPs. With the LC as the key parameter, the formulation was optimized via orthogonal test or single factor screening. We systematically investigated the physicochemical properties, drug loading efficiency, static stability, and acidic media stability of PEG-PLGA/TMP NPs and explored their sustained release behavior under different pH conditions. These NPs were then orally administered at a single dose to rats, and their pharmacokinetic parameters were evaluated and compared with those of free TMP (Figure 1). By developing PEG-PLGA/TMP NPs, this study not only lays the foundation for the clinical application of TMP but also provides new perspectives on enhancing the solubility and bioavailability of drugs with analogous challenges. These NPs potentially serve as a promising alternative to existing TMP dosage forms, optimizing its therapeutic effects.

## 2. Materials and Methods

### 2.1. Materials

Trimethoprim (purity > 98.0%) was purchased from Tokyo Chemical Industry Co., Ltd. (TCI, Tokyo, Japan). Poly(D,L-lactide-co-glycolide) (PLGA; Resomer^®^RG 504H, lactide–glycolide ratio 50:50, acid terminated, Mw 38,000–54,000), polyethylene glycol (PEG; Mw 5500–7500), polyvinyl alcohol (PVA; Mw 13,000–23,000), and hydrochloric acid (HCl) were procured from Sigma-Aldrich (St. Louis, MO, USA). Dichloromethane (DCM) was supplied by Tianjin Xinbote Chemical Industry Co., Ltd. (Tianjin, China). For UPLC and ultra-performance liquid chromatography-tandem mass spectrometry (UPLC-MS/MS) analysis, grade solvents such as methanol (MeOH) and formic acid were purchased from Fisher Chemicals (Newington, NH, USA). Phosphate-buffered saline (PBS) was obtained from Bioss antibodies (Beijing, China). Dialysis membranes (MW cutoff 8000–14,000 Da) were provided by Solarbio^®^ Life Sciences (Beijing, China). All experiments were conducted using ultrapure water obtained from a Milli-Q system from Millipore^®^.

### 2.2. Preparation of PEG-PLGA/TMP NPs by Emulsification Method

PEG-PLGA/TMP NPs were synthesized using a single-emulsion O/W solvent evaporation method [25]. TMP (39 mg), PEG (97.5 mg), and PLGA (390 mg) were separately dissolved in DCM. The TMP solution was added dropwise to the PEG solution under continuous stirring, followed by sonication at 300 W (pulse cycle: 4 s on/ 2 s off) for 1 min. Under ice-water bath conditions, the mixture was slowly added to the PLGA while being magnetically stirred to form the organic phase. The aqueous phase was a 0.5% (*w*/*v*) PVA solution with a volume of 10 mL. The organic phase was combined with the aqueous phase in an ice-water bath and emulsified by intermittent sonication at 300 W (pulse cycle: 4 s on/ 2 s off) for 5 min. Next, the organic solvent was rapidly removed by evaporation under vacuum at 37 °C for 10 min. Finally, the suspension was washed three times to remove unentrapped drug and collected by centrifugation at 20,000 rcf for 30 min at 4 °C. The collected pellets were redispersed in distilled water, sonicated for 10 min to produce free-flowing NPs, which were then frozen at −80 °C and lyophilized for 24 h. TMP NPs were prepared using the same method but without nanocarriers.

### 2.3. Characterization of PEG-PLGA/TMP NPs

#### 2.3.1. Particle Size, PDI, Zeta Potential, and Scanning Electron Microscopy (SEM)

The particle size, PDI, and zeta potential of PEG-PLGA/TMP NPs were measured in triplicate using dynamic light scattering (Zetasizer Nano S90, Malvern Instruments Ltd., Malvern, UK). The samples were diluted with ultrapure water and analyzed at 25 °C with a detection angle of 90°. For morphological characterization, freeze-dried NPs were placed on aluminum sample holders and then gold-plated. Measurements were carried out on a Gemini 360 field emission scanning electron microscope (Zeiss, Jena, Germany) at an acceleration voltage of 5–15 kV.

#### 2.3.2. Fourier Transform Infrared Spectroscopy (FTIR)

FTIR analysis was performed on an IRTracer-100 spectrometer (Shimadzu Ltd., Kyoto, Japan). Samples were prepared by separately mixing 2 mg of TMP, PEG, PLGA, or PEG-PLGA/TMP NPs with 200 mg of potassium bromide (KBr) using a standard pellet method. Spectra were acquired in the wavenumber range of 400 to 4000 cm^−1^ with a resolution of 4 cm^−1^ (32 scans per sample).

#### 2.3.3. Differential Scanning Calorimetry (DSC) Analysis

DSC analysis was used to characterize the thermal behavior of every pure component, TMP, PLGA, PEG, and PEG-PLGA/TMP NPs with differential thermal analyzer (Netzsch Co., Ltd., Selb, Germany). Samples weighing 3–5 mg were placed in flat-bottomed aluminum pans and heated from 30 to 300 °C at a constant rate of 10 °C/min under a nitrogen atmosphere.

#### 2.3.4. Powder X-Ray Diffraction (PXRD) Analysis

XRD patterns of the lyophilized PEG-PLGA/TMP NPs powder, pure TMP, PLGA, PEG, and their physical mixture were characterized using an X-ray powder diffractometer (Bruker Co., Ltd., Karlsruhe, Germany, Model: D8 Advance) equipped with CuKα radiation. The diffraction data were collected at a scanning rate of 5° min^−1^ over a 2θ angular angle of 5–80° at room temperature.

### 2.4. LC and EE of PEG-PLGA/TMP NPs

The LC and EE of PEG-PLGA/TMP NPs were determined by UPLC according to the method described in the literature [26]. Analyses were carried out on Agilent 1290 UPLC system (Agilent Technologies Corp., Santa Clara, CA, USA) equipped with a diode array detector (DAD) and an Agilent ZORBAX Eclipse Plus-C18 column (4.6 × 250 mm, 5 µm). The mobile phase consisted of 0.1% formic acid in water (A) and 0.1% formic acid in methanol (B), with a gradient elution performed as detailed in Table 1. Chromatographic conditions included a flow rate of 1 ml/min, injection volume of 5 µL, detection wavelength of 280 nm, and column temperature maintained at 30 °C. Before the sample injection, all solutions were filtered through a Nylon 66 membrane filter (0.45-μm pore size, Millipore, Burlington, MA, USA).

The supernatant from the centrifuged and washed nanosuspensions (as described in Section 2.2) was collected. The content of free TMP in the supernatant was determined using the aforementioned UPLC method, and the EE was then calculated.

The lyophilized powder of PEG-PLGA/TMP NPs was accurately weighed. The EE and LC were then calculated using the following Formulas (1) and (2):

(1) $$EE\%=\frac{W_{total}-W_{free}}{W_{total}}\times 100\%$$

(2) $$LC\%=\frac{W_{total}-W_{free}}{W_{dried}}\times 100\%$$

where W_total_, W_free_, and W_dried_ represented the total mass of TMP, the mass of free TMP quantified in the supernatant, and the mass of lyophilized PEG-PLGA/TMP NPs powder, respectively.

### 2.5. Stability of PEG-PLGA/TMP NPs

To evaluate the stability of newly formulated lyophilized PEG-PLGA/TMP NPs, their particle size, PDI, and zeta potential were measured at 1, 4, 7, 10, 15, and 30 days after being stored at room temperature (25 °C) or under refrigeration (4 °C). On the other hand, the EE of the NPs was measured at different time points (0, 1, 2, 3, and 4 h) when they were placed in HCl solution with a pH of 1.2.

### 2.6. In Vitro Release of PEG-PLGA/TMP NPs

The in vitro drug release of TMP from PEG-PLGA/TMP NPs was measured using a previously described method [27]. Briefly, lyophilized PEG-PLGA/TMP NPs powder (equivalent to 3 mg TMP) was dispersed in 10 mL of pH 1.2 HCl or pH 6.8 PBS, and the dispersion was transferred into dialysis membranes (MW cutoff 8000–14,000 Da). These dialysis membranes were then submerged in 300 mL of corresponding HCl or PBS medium at 37 °C, agitated continuously at 125 rpm. At predetermined time points (0.25, 0.5, 1, 2, 4, 6, 8, 10, 12, and 24 h), 1 mL sample were withdrawn from the external dialysis medium for measurement, and an equal volume of fresh medium was added to maintain constant volume. All experiments were performed in triplicate, and the released TMP concentration in the external medium was quantified by UPLC-MS/MS to generate the cumulative release curve.

### 2.7. Evaluation of the Ex Vivo and In Vivo Relevance of PEG-PLGA/TMP NPs

The in vitro–in vivo correlation (IVIVC) of the drug was calculated using the Wagner–Nelson method, with the in vitro cumulative release rate at pH 6.8 set as the independent variable and the in vivo absorption rate as the dependent variable. The cumulative release rate and in vivo absorption rate were collected at 0.5, 1, 2, 4, 6, 8, and 12 h.

(3) $$In\;vivo\;absorption\left(\%\right)=\frac{C_{t}+K_{e}\times {AUC}_{0-t}}{K_{e}\times {AUC}_{0-\infty }}$$

The calculation was based on Formula (3), where C_t_ represents the drug concentration at a certain time point (t), K_e_ is the elimination rate constant, AUC_0−t_ is the area under the drug concentration-time curve from time zero to t, and AUC_0−∞_ is the area under the drug concentration-time curve from time zero to infinity [28].

### 2.8. Animals

Male Sprague-Dawley (SD) rats, weighing 180 ± 10 g, were sourced from the Laboratory Animal Center of Lanzhou Veterinary Research Institute (Lanzhou, China). The pharmacokinetic test was approved by the Animal Ethics Committee of Lanzhou Institute of Husbandry and Pharmaceutical Sciences.

### 2.9. UPLC-MS/MS Analysis

The concentration of TMP in plasma and the in vitro drug release were determined using UPLC-MS/MS. The analysis was performed on an ExionAD system (AB SCIEX Corp., Framingham, MA, USA), equipped with an electrospray ionization (ESI) source and a Triple Quad^TM^ 3500 mass spectrometer (AB SCIEX Corp., Framingham, MA, USA). Data acquisition and processing were conducted using OS Analyst software (Version: 1.7.0.36606, AB SCIEX Corp., Framingham, MA, USA). The Agilent InfinityLab Poroshell 120 EC-C18 column (4.6 × 100 mm, 2.7 µm) was employed as the chromatography column at a temperature of 30 °C with mobile phase consisting of 0.1% formic acid water (A) and 0.1% formic acid in methanol (B). The gradient elution process was set as below: from 0 to 1 min, the mobile phase consisted of 80% A; from 7 to 8 min, it was 20% A; and from 9 to 13 min, it returned to 80% A. The flow rate was set at 0.5 mL/min, and the injection volume was 5 µL. Detection was carried out using electrospray ionization (ESI). Quantitative detection was performed in the multi-ion reaction monitoring (MRM) mode. In the positive ion mode, the capillary voltage was set to +4500 V, the ion source temperature was 550 °C, the nebulizing gas pressure was 55 psi, the auxiliary gas pressure was 55 psi, the curtain gas was 35 psi, and the dwell time was 100 ms.

### 2.10. Pharmacokinetic Study

#### 2.10.1. Pharmacokinetic Assay

Twelve male SD rats were randomly assigned to two groups (n = 6): the free TMP group and the PEG-PLGA/TMP NPs group. All rats were acclimatized under standard laboratory conditions with free access to food and water for 5 days, followed by a 12 h fasting period (water allowed) prior to drug administration. Subsequently, corresponding drugs were administered by oral gavage, with the dosage calculated based on 20 mg/kg of TMP. Blood samples (500 µL) were collected from the orbital plexus at scheduled time points (0.083, 0.25, 0.5, 1, 2, 4, 6, 8, 12, 24, 36, 48, and 72 h) into heparinized centrifuge tubes.

#### 2.10.2. Sample Preparation

Plasma was extracted by centrifuging the blood samples at 3000 rpm for 10 min at 25 °C. Then, 400 µL of methanol was added to 200 µL of plasma sample to remove proteins, and the mixture was centrifuged at 12,000 rpm for 10 min at 25 °C. The resulting supernatant was filtered through 0.22 µm syringe filters (Millipore) and transferred to an injection vial for subsequent analysis by UPLC-MS/MS.

#### 2.10.3. Data Preparation

Pharmacokinetic parameters were determined via the plasma (200–202) implementation of the non-compartmental model embedded in the pharmacokinetic analysis module of Phoenix WinNonlin 8.3. With this non-compartmental approach, parameters such as peak plasma concentration (C_max_), the time at which peak plasma concentration occurred (T_max_), the area under the curve of plasma drug concentration versus time (AUC_0−t_), elimination half-life (T_1/2_), mean residence time (MRT), apparent volume of distribution (V_z_), and clearance (CL) were determined.

### 2.11. Statistical Analysis

Data are presented as the Mean ± standard deviation (S.D.) and analyzed using SPSS software (version 27.0, IBM, New York, NY, USA). One-way ANOVA was used to determine statistical significance, with a *p*-value of 0.05 considered significant.

## 3. Results and Discussion

### 3.1. Optimization of PEG-PLGA/TMP NPs

Considering the practicability of oral administration, the LC was selected as the most crucial factor for the orthogonal experiment design aiming to optimize the formulation of PEG-PLGA/TMP NPs [26]. Four key variables, namely the TMP concentration, PVA concentration, sonication power, and centrifugation speed, were systematically investigated. After defining the variables and their corresponding levels (Table 2), the orthogonal test was constructed to identify the optimal preparation parameters. Orthogonal analysis revealed the following order of influence on LC: TMP concentration > sonication power > centrifugation speed > PVA concentration (Table 3). The optimal formulation comprised a TMP concentration of 3 mg/mL, PVA concentration of 0.5%, sonication power of 300 W, and a centrifugation speed of 20,000 rcf.

### 3.2. Physicochemical Properties

**Figure 2:**
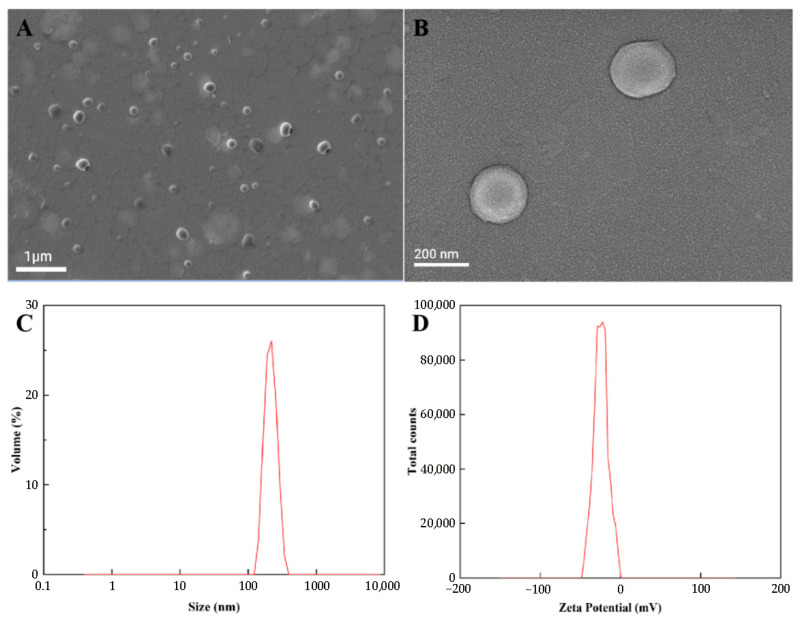
Characteristics of the PEG-PLGA/TMP NPs: (**A**,**B**) SEM images, (**C**) Size distribution, (**D**) Zeta potential.

The surface morphologies of the obtained samples were investigated by SEM. As shown in Figure 2A,B, the PEG-PLGA/TMP NPs were found to be spherical in shape, with a narrow size distribution and particle sizes ranging from 200 to 300 nm. Dynamic light scattering measurements of the optimized formulation showed a mean particle size of 245 ± 40 nm (Figure 2C), which was consistent with the result of SEM. A low PDI of approximately 0.1 indicated that the PEG-PLGA/TMP NPs were monodisperse and hardly exhibited any particle aggregation [29]. Additionally, the mean zeta potential, EE, and LC of the PEG-PLGA/TMP NPs were measured to be −23.8 ± 1.2 mV (Figure 2D), 88.2 ± 4.3%, and 34.0 ± 1.6%, respectively, as presented in Table 4. The high drug LC far exceeds that of most reported TMP nanocarriers [10], demonstrating the synergistic advantage of PEG-PLGA matrix properties and the optimized emulsion solvent evaporation method. The mean zeta potential of −23.8 ± 1.2 mV indicated that the surface of NPs is negatively charged, which is crucial for maintaining NPs stability through electrostatic repulsion. This can ensure minimal particle aggregation and monodispersity of NPs in biological media, consistent with the low PDI results [30]. It is worth mentioning that PEG-PLGA, known for its suitability for freeze-drying, shows a high drug EE when entrapping drugs with low water solubility [31].

**Figure 3:**
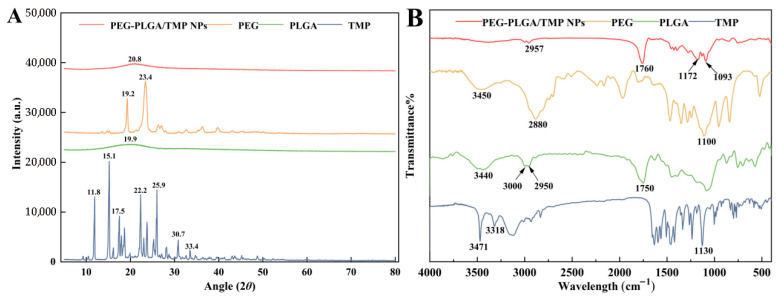
Characteristics of the PEG-PLGA/TMP NPs: (**A**) PXRD, (**B**) FTIR.

PXRD was used to analyze the degree of crystallinity and the physical state of the PEG-PLGA/TMP NPs and their individual components (Figure 3A). The PXRD patterns provided strong evidence for the successful formulation of PEG-PLGA/TMP NPs. TMP presented sharp and intense peaks at diffraction angles of 11.8°, 15.1°, 17.5°, 22.2°, 25.9°, 30.7°, and 33.4°, which suggested that the drug existed in a crystalline state [32]. PLGA showed a dome-shaped region in the 2θ range from 15° to 25° because of its amorphous state [33]. The diffractogram of PEG exhibited two peaks at 19.2° and 23.4°, which were indicative of its crystallinity [25]. To investigate whether lyophilization itself could induce TMP amorphization, we conducted a control experiment where pure TMP was lyophilized under identical conditions as the NPs. PXRD analysis of the lyophilized TMP revealed persistent crystalline peaks at 11.8°, 15.1°, and 22.2° (Figure S1), confirming that lyophilization alone did not cause amorphization. In contrast, the PXRD pattern of the PEG-PLGA/TMP NPs lacked any TMP-related diffraction peaks, clearly indicating that TMP existed in an amorphous state within the NPs, a result attributed to strong interactions between the drug and the polymeric matrix. The FTIR spectra of the PEG-PLGA/TMP NPs and their individual component are presented in Figure 3B. The characteristic peaks of TMP at 3471 cm^−1^ and 3318 cm^−1^, corresponding to the -NH_2_ stretching vibration peaks and at 1130 cm^−1^, belong to the C-O stretching vibration peak disappeared in the PEG-PLGA/TMP NPs [32], suggesting a disruption in the intermolecular hydrogen bonding of the TMP drug. Moreover, several new peaks were detected in the PEG-PLGA/TMP NPs, including C-H stretching (2957 cm^−1^) from PEG-PLGA, a shifted C=O stretching band (1760 cm^−1^ vs. 1750 cm^−1^ of PLGA), and C-O-C vibrations (1172 cm^−1^ and 1093 cm^−1^) [34], collectively indicating hydrogen bonding between TMP and the ester carbonyl groups of PLGA. These results demonstrated the successful amorphous encapsulation of TMP within PEG-PLGA NPs through intermolecular interactions.

**Figure 4:**
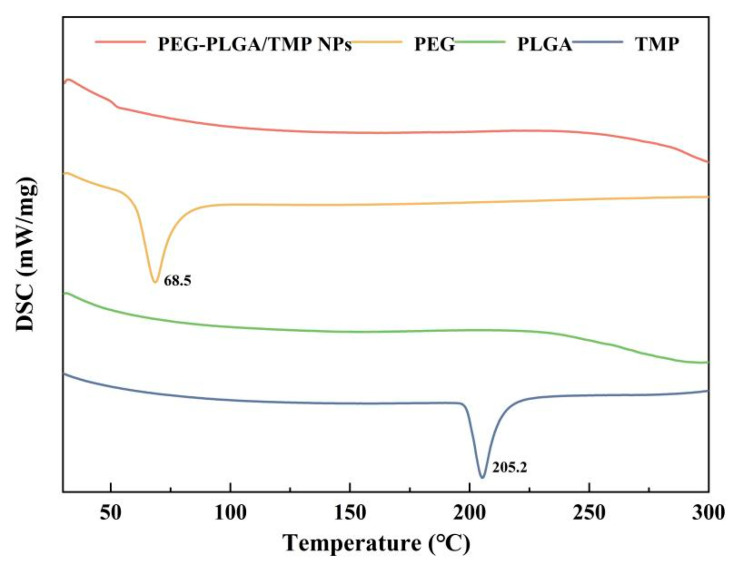
DSC thermograms of PEG-PLGA/TMP NPs.

To further determine the physical state of TMP in the NPs based on thermal characterization, DSC analysis was performed (Figure 4). An endothermic peak of TMP was observed at 205.2 °C, which corresponds to its melting point [35]. Notably, the absence of the TMP melting peak in the DSC thermogram of PEG-PLGA/TMP NPs indicated the amorphous dispersion of TMP within the polymeric matrix, which was in accordance with the XRD results. Significantly, the molecular dispersion of TMP in this form can effectively facilitate its dissolution and intestinal absorption, ultimately contributing to the enhancement of its bioavailability.

### 3.3. Static Stability and Stability in Acidic Media

**Figure 5:**
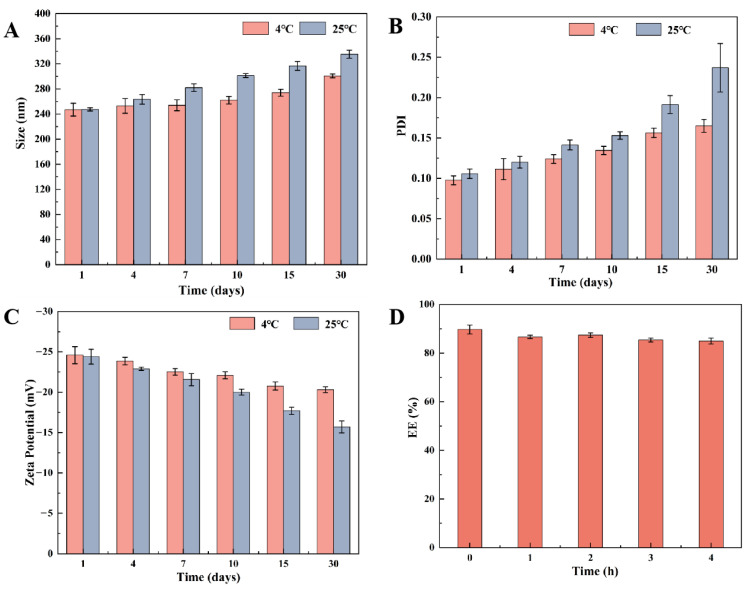
(**A**–**C**) Variations in particle size, PDI, and zeta potential of PEG-PLGA/TMP NPs during one month storage at room temperature or under refrigeration, (**D**) EE change of PEG-PLGA/TMP NPs in acidic media (pH 1.2), (n = 3).

During the storage of nanoformulations, NPs are prone to aggregation, resulting in heterogeneous particle size distribution, which generally poses significant challenges to the absorption process of nanomedicines [36]. To study the stability of PEG-PLGA/TMP NPs, their particle size, PDI, and zeta potential, changes were investigated at various temperatures (4 °C and 25 °C) over 30 days (Figure 5A–C). At 4 °C, the PEG-PLGA/TMP NPs maintained stable physicochemical properties, with particle sizes ranging from 240 to 300 nm, PDI consistently below 0.16, and zeta potential absolute values below 20 mV. Comparatively, storage at 25 °C showed increased fluctuations in all three parameters, demonstrating reduced stability at room temperature. These results strongly suggested that lyophilized PEG-PLGA/TMP NPs should be stored in a refrigerator. The observed fluctuations at 25 °C highlight a potential challenge for real-time stability during transportation or handling outside refrigeration. Future studies will validate long-term stability and scale-up feasibility to bridge the lab-to-clinic translation gap. Additionally, the EE of PEG-PLGA/TMP NPs showed no significant change in acidic media (Figure 5D), which implies that the NPs may maintain good stability in the gastric fluid environment, thus ensuring their structural integrity.

### 3.4. In Vitro Release

**Figure 6:**
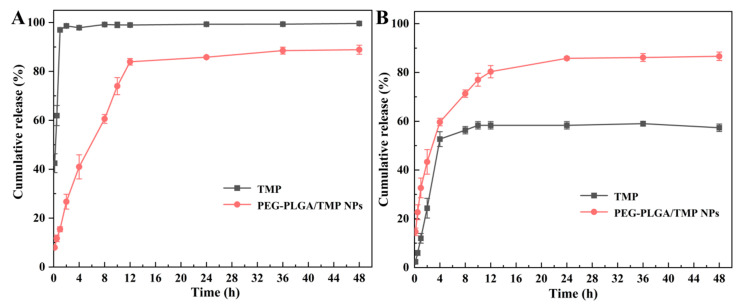
(**A**) The accumulation release profiles of PEG-PLGA/TMP NPs at 37 °C in HCl solution (pH 1.2) and (**B**) in PBS (pH 6.8), with n = 3.

To investigate the effect of PEG-PLGA modification on the release behavior of TMP, we compared the 48 h release profiles of PEG-PLGA/TMP NPs and free TMP at 37 °C. In a pH 1.2 medium, free TMP rapidly released 96.97% of its amount within 1 h, while PEG-PLGA/TMP NPs demonstrated remarkable sustained release properties (Figure 6A). The release of TMP from the NPs at pH 1.2 exhibited a biphasic pattern as follows: an initial rapid release of approximately 70–75% of the total TMP occurring within the first 10 h, followed by a slower release phase. As commonly observed in PEG-PLGA systems, the burst phase occurs because the swelling of the surface layer promotes the detachment of drugs, and the significant concentration gradient on the surface drives the rapid diffusion of drugs [37]. Critically, the PEG-PLGA significantly delayed TMP degradation in acidic media, aligning with its demonstrated stability under low-pH conditions. Notably, in pH 6.8 PBS, the cumulative release rate of TMP was only 58% over 48 h, while that of PEG-PLGA/TMP NPs reached 86%, suggesting that PEG-PLGA promotes TMP release at this pH (Figure 6B). Significantly, under both pH conditions, the sustained release performance of PEG-PLGA/TMP NPs outperformed that of free TMP, indicating that the designed PEG-PLGA/TMP NPs can be effectively delivered to the intestine in an essentially intact form. Consequently, we further speculate that PEG-PLGA/TMP NPs can enhance the oral bioavailability of TMP.

Furthermore, we employed multiple kinetic models, including zero order, first order, Higuchi, and Korsmeyer–Peppas equations, to evaluate the dissolution profiles of PEG-PLGA/TMP NPs. The mathematical models, equations, and correlation coefficients were shown in Table 5. The release of PEG-PLGA/TMP NPs was best described by a first-order model (pH 1.2: R^2^ = 0.9886, pH 6.8: R^2^ = 0.9623), suggesting that the release process depends on the TMP concentration. Korsmeyer–Peppas analysis further elucidated the mechanism: release index (n = 0.41 at pH 1.2; n = 0.23 at pH 6.8) confirmed Fickian diffusion dominance, consistent with previously reported diffusion-controlled release mechanisms for PEG-PLGA systems [38].

### 3.5. In Vivo Pharmacokinetic Evaluation

**Figure 7:**
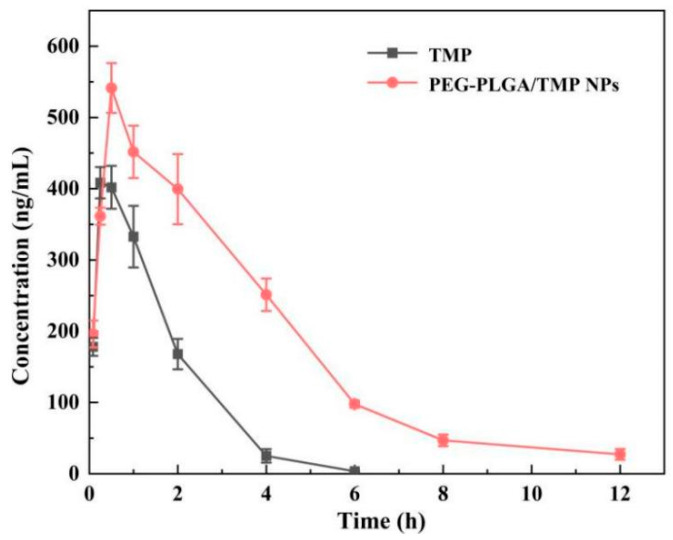
Plasma drug concentrations of oral TMP and PEG-PLGA/TMP NPs in rats (n = 6).

This pharmacokinetic research aimed to evaluate the impact of PEG-PLGA polymeric NPs on the bioavailability and pharmacokinetic behavior of orally administered TMP compared to its free form. The plasma concentration-time curves after administration with a dosage of TMP at 20 mg/kg are presented in Figure 7, and the pharmacokinetic parameters were also summarized in Table 6. After intragastric administration of PEG-PLGA/TMP NPs, the TMP in plasma swiftly reached the peak concentrations of 541.33 ± 35.00 ng/mL at 0.5 h, then decreased gradually and sustained above 30 ng/mL for 12 h. Comparatively, free TMP more quickly reached the peak value of 417.51 ± 23.34 ng/mL at 0.33 ± 0.13 h, and then declined rapidly to 30 ng/mL within 4 h after intragastric administration. Pharmacokinetic parameters further highlighted the superiority of the NPs: the AUC_0−∞_, t_1/2_, and MRT of the NPs were 2298 ± 227 ng·h/mL, 2.47 ± 0.19 h, and 3.10 ± 0.11 h, respectively, whereas for free TMP, these parameters were 814 ± 99 ng·h/mL, 0.72 ± 0.08, and 1.27 ± 0.11 h, respectively, Compared with free TMP, the bioavailability, t_1/2_, and MRT of the prepared PEG-PLGA/TMP NPs increased 2.82-, 3.43-, and 2.44-fold, respectively, demonstrating unique advantages in long-acting drug release compared to existing TMP formulations. These results indicated that the prepared NPs exhibited a sustained release effect and improved the oral bioavailability of TMP.

These results demonstrated that PEG-PLGA NPs not only prolonged systemic exposure through sustained release but also significantly improved oral bioavailability. The enhanced performance may be attributed to three synergistic mechanisms. Firstly, TMP was dispersed in the NPs in an amorphous form, circumventing the need for crystalline drugs to overcome lattice energy for dissolution, which significantly enhanced its solubility and dissolution rate, thus allowing more drug to be absorbed into the bloodstream. Secondly, the modification with the hydrophilic polymer PEG enhanced the dispersibility and stability of the NPs. Moreover, PEG reduced the likelihood of NPs being recognized and phagocytosed by the reticuloendothelial system [39], thereby extending the circulation time of NPs in the body. Finally, according to previous stability test results, PEG-PLGA/TMP NPs could maintain stability in acidic solutions, protecting TMP from degradation in the gastric acid environment and ensuring that the drug could reach the intestinal absorption site intact. Collectively, these features facilitated nanoparticle transport across epithelial barriers via transcellular, paracellular, and lymphatic pathways, effectively overcoming the absorption limitations of poorly soluble TMP and ultimately enhancing oral bioavailability. Additionally, it is noteworthy that compared with the rapidly cleared free TMP, the prolonged maintenance of drug concentration achieved by PEG-PLGA/TMP NPs has laid a solid pharmacokinetic foundation for enhancing in vivo antibacterial activity.

### 3.6. Evaluation of Ex Vivo and In Vivo Relevance

**Figure 8:**
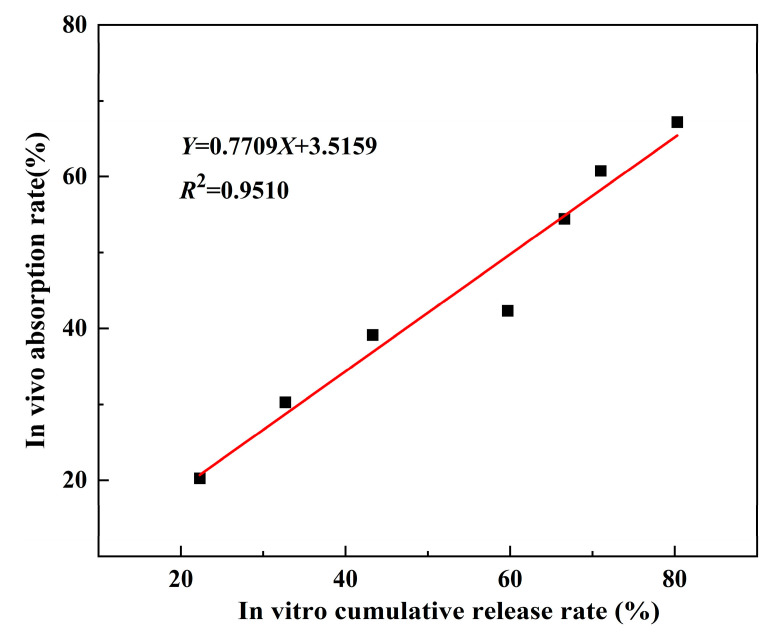
Correlation between in vitro cumulative release and in vivo absorption rates of PEG-PLGA/TMP NPs.

The in vitro and in vivo correlations of PEG-PLGA/TMP NPs are depicted in Figure 8. A proportional relationship was observed between cumulative in vitro drug release and systemic absorption, with the latter increasing synchronously as the former increased. According to the literature [40], a coefficient of determination (R^2^) between 0.49 and 0.80 indicates a strong correlation, while an R^2^ between 0.81 and 1 represents a very strong correlation. Therefore, it can be concluded that there is a very strong correlation between the in vivo absorption and in vitro release of PEG-PLGA/TMP NPs. This robust correlation confirms that in vitro release profiles reliably predict in vivo absorption kinetics of TMP, providing valuable guidance for the optimal design of TMP nanoformulation. By strategically adjusting excipient ratios and fabrication parameters based on in vitro release data, researchers can systematically enhance bioavailability and achieve desired therapeutic outcomes.

## 4. Conclusions

In this study, PEG-PLGA NPs were successfully developed using an emulsion solvent evaporation method as an effective oral delivery system for TMP to enhance its solubility and bioavailability. Through orthogonal experimental optimization, the optimized formulation achieved a high drug LC of 34.0 ± 1.6% and EE of 88.2 ± 4.3%, with a uniform particle size of 245 ± 40 nm, and low PDI of 0.103 ± 0.019. Notably, this LC is significantly higher than existing literature reports, serving as the core innovation to overcome the clinical application bottleneck of nanocarriers. These NPs exhibited excellent stability, maintaining stable physicochemical properties over 30 days when stored at 4 °C and showing no significant change in EE in acidic media, which suggests structural integrity in the gastric fluid environment. In vitro release studies showed PEG-PLGA/TMP NPs had sustained release properties with a biphasic pattern in pH1.2 and promoted release in pH6.8, following a first-order model with Fickian diffusion. Their amorphous TMP state, confirmed by PXRD and DSC analyses, enabled 86% cumulative release at pH 6.8 over 48 h. In vivo pharmacokinetic studies demonstrated that PEG-PLGA/TMP NPs significantly prolonged the t_1/2_ of TMP to 2.47 ± 0.19 h compared to 0.72 ± 0.08 h observed with free TMP, extended the MRT from 1.27 ± 0.11 h to 3.10 ± 0.11 h, and improved oral bioavailability 2.82-fold. This significant prolongation of t_1/2_ and MRT confirms the long-acting release characteristics of the NPs, which is attributed to their prolonged systemic circulation and reduced gastric degradation. The robust in vitro–in vivo correlation demonstrated by an R^2^ value of 0.9623 further validated the design of PEG-PLGA/TMP NPs for achieving predictable and reliable therapeutic outcomes. These findings suggest that the PEG-PLGA/TMP NPs not only address the inherent limitations of TMP, such as poor solubility and rapid clearance, but also enhance its oral bioavailability. These advancements highlight the potential of PEG-PLGA/TMP NPs as a promising alternative to conventional formulations. In clinical practice, the improved oral bioavailability and prolonged half-life of TMP may allow for reduced dosing frequency (e.g., from twice daily to once daily), thereby enhancing patient treatment compliance. In the veterinary field, this formulation may address the urgent need for effective TMP delivery in livestock infections, offering promise for improving therapeutic outcomes.

Future studies on the PEG-PLGA/TMP NPs should prioritize comprehensive safety evaluations, including chronic toxicity assessments, and explore their synergistic antibacterial effects with other antimicrobial agents in infection models. This will better delineate their enhanced therapeutic advantage over conventional TMP formulations currently used in clinical practice. Additionally, potential challenges related to large-scale production and batch reproducibility should be systematically addressed before advancing toward clinical applications.

## Figures and Tables

**Table 1:** The UPLC gradient elution method for determining the LC and EE of PEG-PLGA/TMP NPs.

Time (min)	Mobile Phase A	Mobile Phase B
0	80	20
1	80	20
7	20	80
8	20	80
9	80	20
13	80	20

**Table 2:** Factors and levels of the L9 (3^4^) orthogonal design.

Levels	Variables			
	Concentration (TMP, mg/mL)	Concentration (PVA, %)	Sonication Power (W)	Centrifugation Speed (rcf)
1	1.5	0.5	200	14,000
2	3	1	250	16,000
3	4	2	300	20,000

**Table 3:** The optimization of PEG-PLGA/TMP NPs by orthogonal experiment.

Sample	Concentration (TMP, mg/mL)	Concentration (PVA, %)	Sonication Power (W)	Centrifugal Speed (rcf)	LC (%)
1	1	1	1	1	10.4 ± 0.2
2	1	2	2	2	10.9 ± 0.3
3	1	3	3	3	17.5 ± 0.6
4	2	1	2	3	34.0 ± 1.6
5	2	2	3	1	26.3 ± 1.1
6	2	3	1	2	15.6 ± 0.4
7	3	1	3	2	22.8 ± 1.3
8	3	2	1	3	14.6 ± 0.4
9	3	3	2	1	20.0 ± 1.2
K_1_	38.8	67.2	40.6	56.7	
K_2_	75.9	51.8	64.9	49.3	
K_3_	57.4	53.1	66.6	66.1	
k_1_	12.9	22.4	13.5	18.9	
k_2_	25.3	17.3	21.6	16.4	
k_3_	19.1	17.7	22.2	22.0	
R	12.4	5.1	8.7	5.6	
Optimum	2	1	3	3	

Notes: K_1_, K_2_, and K_3_ represents the sum of the LC under different levels of a certain factor. k_1_, k_2_, and k_3_ are the average values of the K_1_, K_2_, and K_3_ respectively. R represents the range value, calculated by taking the difference between the maximum and minimum values among k_1_, k_2_, and k_3_.

**Table 4:** Physicochemical characterization of PEG-PLGA/TMP NPs.

Particle Size (nm)	PDI	Zeta Potential (mV)	LC (%)	EE (%)
245 ± 40	0.103 ± 0.019	−23.8 ± 1.2	34.0 ± 1.6	88.2 ± 4.3

**Table 5:** Release kinetic parameters of PEG-PLGA/TMP NPs fitted to various kinetic models.

	pH 1.2		pH 6.8	
	Equation	R^2^	Equation	R^2^
Zero order	Q=15.46+2.52t	0.8048	Q=47.64+1.27t	0.5150
First order	$Q=86.96(1-e^{-0.21})$	0.9886	$Q=85.13(1-e^{-0.30})$	0.9623
Higuchi	$Q=16.29t^{1/2}+3.17$	0.9408	$Q=10.95t^{1/2}+28.82$	0.7785
Korsmeyer-Peppas	$Q=22.89t^{0.41}$	0.9534	$Q=40.01t^{0.23}$	0.9106

**Table 6:** In vivo pharmacokinetic parameters of oral TMP and PEG-PLGA/TMP NPs in rats (n = 6).

Parameter	Unit	TMP	PEG-PLGA/TMP NPs
AUC_0−t_	h·ng/mL	802 ± 104	2134 ± 188 *
AUC_0−∞_	h·ng/mL	814 ± 99	2298 ± 227 *
C_max_	ng/mL	417.51 ± 23.34	541.33 ± 35.00 *
T_max_	h	0.33 ± 0.13	0.50 *
t_1/2_	h	0.72 ± 0.08	2.47 ± 0.19 *
MRT	h	1.27 ± 0.11	3.10 ± 0.11 *
Vd	L/kg	25.65 ± 3.29	31.97 ± 2.51 *
CL	L/h/kg	24.87 ± 3.26	9.03 ± 0.85 *
F (%)	/	/	282.31%

Note: * Statistical significance compared with TMP is *p* < 0.01. Abbreviations: AUC_0−t_ indicates the area under the curve from time zero to t; AUC_0−∞_ represents the area under the curve from time zero to infinity; C_max_ stands for the maximum plasma concentration, with T_max_ denoting the time taken to reach this maximum value; t_1/2_ is the elimination half-life; MRT is the mean residence time; Vd represents the volume of distribution; CL is the body clearance; and F is the relative bioavailability.

## Data Availability

Data will be made available on request.

## Supplementary Materials

The following supporting information can be downloaded at: https://www.mdpi.com/article/10.3390/pharmaceutics17080957/s1, Figure S1. PXRD diffraction spectra of lyophilized TMP.
