# Supplementary figures and images for: Enhancing the Solubility and Oral Bioavailability of Trimethoprim Through PEG-PLGA Nanoparticles: A Comprehensive Evaluation of In Vitro and In Vivo Performance
Source: Pharmaceutics. 2025 Jul 24;17(8):957. doi: 10.3390/pharmaceutics17080957 (PMC12389140; doi:10.3390/pharmaceutics17080957)

## Supporting Information

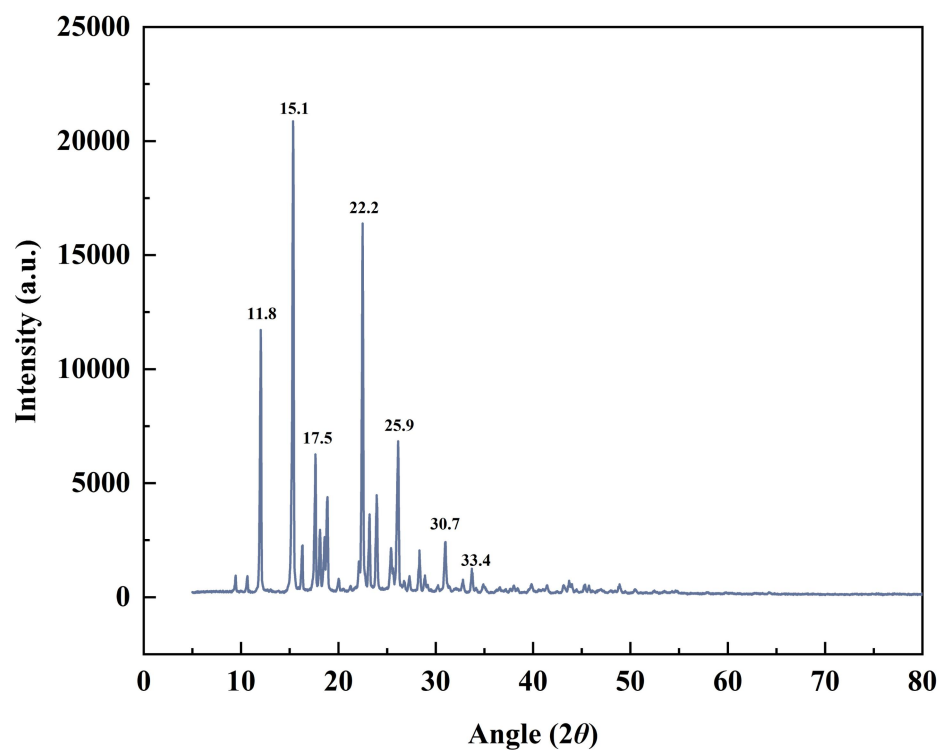

**Figure S1.** PXRD diffraction spectra of lyophilized TMP.

Supplement: Supplementary file 1 [file pharmaceutics-17-00957-s001.zip › pharmaceutics-3715655-supplementary.pdf]
